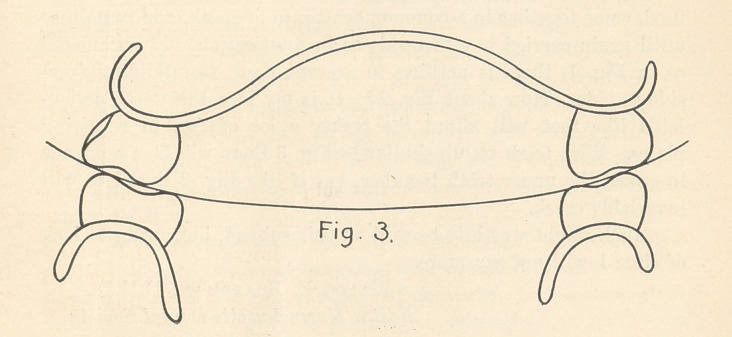# Massachusetts Dental Society

**Published:** 1903-04

**Authors:** 

**Affiliations:** Massachusetts Dental Society


### MASSACHUSETTS DENTAL SOCIETY.

(Continued from page 221.)

THURSDAY morning at nine o'clock the clinics were opened, and consisted of the following:

1. Joseph Head, M.D., D.D.S., Philadelphia, Pa. (*a*) A simple method of gold-plating. (*b*) Porcelain inlays.

2. Charles C. Patten, D.D.S., Boston, Mass. On Jenkins's Body; Hammond Furnace.

3. Dr. Julius F. Hovestadt, Boston, Mass. Porcelain tips and restoring contours by means of porcelain.

4. Mr. Leonard A. Jenkins, Portland, Me. Exhibition and clinic of the Dr. Jenkins's porcelain inlay outfit and work.

5. Charles D. Meeker, D.D.S., Newark, N. J. Manipulation of the archite cement.

6. Walter F. Bisbee, D.D.S., Camden, Me. Crown- and bridge-work. Dr. W. H. Baird's method of making crowns.

7. Dr. Thomas D. Shumway, Plymouth, Mass. Combination tin and gold fillings.

8. Edward Page, M.D., D.M.D., Boston, Mass. Test for amalgam, relative to strength, color, shrinkage, and expansion.

9. Dr. Frederic Freeman, Boston, Mass., exhibited a working model, on skull, of a mechanical appliance supplying the loss of one-half of the inferior maxilla with ball-and-socket joint.

10. Henry A. Baker, D.D.S., Boston, Mass. An exaggerated case of protrusion of lower jaw reduced without chin-piece or head-gear. Illustrated.

11. Dr. Loomis P. Haskell, Chicago, Ill. Continuous gum-work.

12. Edgar G. Hubbell, Springfield, Mass. An all-gold cuspid crown and contour.

13. Elmer B. Abbey, D.D.S., Hartford, Conn. How to construct a Smith improved crown.

14. Murdock C. Smith, M.D., D.D.S., D.M.D., Lynn, Mass. (*a*) Will have a patient present who has been operated on for giant-cell sarcoma. (*b*) Burnishing gold, and possibly have a patient present.

15. William F. Andrews, D.D.S., Springfield, Mass. The use of metal in repairing vulcanite.

16. Dr. Arthur J. Robinson, Morrisville, Vt. On crown articulating teeth; articulator.

*Dr. Robinson.*—In answer to a letter from your committee asking if I would give a clinic, I wrote your chairman that I had made an all-porcelain crown, using a plain tooth for facing and attaching pin with porcelain body,—by means of a gasoline furnace,—and while I had never seen just the same thing done, I did not claim it was all original, but if after looking over the drawings I was to send him, he found anything worthy, I would be glad to give it at this time. I first prepare the root, then grind tip of tooth to conform to front of root. With a little wax retain it there and get proper articulation, fastening tooth and post together, using wax enough to cover the end of the root. Take an impression of it. Remove tooth, post, and wax and insert another similar post. Take a piece of platinum-foil large enough to cover end of root and a little more, punch a hole in the centre, and place over the post. Then by the use of wax—nearly cold—force the foil into the end of the root. Remove and invert end of post and corners of foil, using any investment you may use for other work. When set remove wax and post and insert post with tooth attached and bring investment up in front of tooth enough to give it support. When investment is sufficiently hardened, pick out wax and if necessary solder post to pins; add porcelain to contour and fuse. The object of this crown is twofold,—first, no time is lost in ordering a crown for a given case; second, no crown can be ground to fit a root as perfectly as can be fitted in this manner of making one. It is also cheaply made and to my mind the strongest crown that can be made of porcelain without a band.

The first advantage may not count much generally with those who live near a supply house, but to us who are working in country places all these things are of interest. For those who are not fortunate in having either gas or a day current of electricity, something must be devised whereby one may get the needed heat and apparatus to fuse the porcelain for crown- and bridge-work and for inlays. As I have neither I cast around until I found a furnace with gasoline as motive power and heat. I refer to the little furnace manufactured by the Turner Brass Works in Chicago, and for sale by many of the supply houses. They also manufacture several other appliances which are useful to the dentist, especially those from the country.

17. Mr. John Hood, Boston, Mass. Waxable rubber.

18. Albert A. Shaw, D.D.S., Cambridgeport, Mass. The Shaw Alloy Machine Company. Why every dentist should use the Shaw alloy machine.

19. Dr. George E. Savage, Worcester, Mass. Table clinics on something new in regulating appliances.

20. Lawrence W. Baker, D.M.D., Boston, Mass. Some attempts at artistic artificial work.

21. John N. Crouse, D.D.S., Chicago, Ill. New cement; also tests of the various cements in use.

Thursday session opened by President Faxon at eleven o'clock.

*President Faxon.*—I am pleased to introduce as the first speaker Dr. L. P. Haskell, of Chicago, Ill.

*Dr. Haskell.*—LADIES AND GENTLEMEN,—It gives me great pleasure to meet the members of the Massachusetts State Dental Society in this city, or rather in the then village of Chelsea, where my dental career was begun fifty-seven years ago. In the mean time I have never before had the pleasure of meeting the dentists of the old Bay State. Those who were practising here in 1856, the year I left Boston, I think have nearly all passed away. Dr. Wetherbee is the only one of the "old guard" I have seen here. I recall the names of Harwood, Parker, Joshua and Elisha Tucker, Wilson, Keep, Leach, Williams, Codman, the Webbers, Stearns, Ball, Morton, Eastman, Cummings, Hitchcock, Flagg, Thresher, Mayo, Salmon, Leseur, Goodno, Hanson, Bartlett, Dickson, Shepard, Noyes, Dickenson. If any of these gentlemen are living, or are present, it would give me great pleasure to meet them.

As for myself, my position in the profession is somewhat unique, from the fact that in all probability there was never another dentist who confined his labors exclusively to the construction of artificial dentures for fifty-seven years. From the outset, although I received instructions in operative dentistry, I determined to pursue the mechanical branch. In those days it was necessary for the student to learn to do some things which in later years have been unnecessary, as, for instance, melting, refining, and rolling gold plate; making solders; grinding up, backing, and soldering full sets of gum teeth; making his own instruments. The limited dental supplies were kept in a drug-store, where was to be found a small stock of teeth, few instruments and appliances, no gold plate, nor solder.

In 1841 I came to Boston and entered a printing-office, to have remained until I was twenty-one, but at the age of nineteen, in 1845, my brother-in-law, Dr. Hanson, urged me to learn dentistry as his student, and I remained with him three years.

While I had instruction in operative dentistry, successfully filling teeth with the non-cohesive gold then used, I devoted my attention to carving and mounting the so-called "block mineral teeth" for our own practice and for the profession, and this work I continued for eleven years.

The opportunities for the dental student were very limited. There was but one dental college, the Baltimore, and it was generally considered that the student could learn dentistry better in the office of a good dentist than in a college. There were no dental journals nor dental societies. Dental offices were close corporations, as the average dentist would not allow other dentists in his operating-room or laboratory. The student had no opportunity of learning anything outside of his preceptor's office.

Were the graduate of to-day to be placed in an old-time office, with the methods, appliances, and materials of those days, and told to perform any operation, operative or mechanical, he would be at a loss to know how to do it.

In 1856 I removed with my preceptor to Milwaukee, remaining one year, and then located in Chicago, where I have since resided.

I come before you this morning with no theories, no fads, but simply to tell you what I have learned from experience, a plain, unvarnished tale. There are many theories put forth in the dental journals. For instance, there is the theory of the expansion of plaster, which is true, but I have never found, however, that it interferes with the fit of the plate. In 1859 I came to Boston on a visit. Calling on my old friend Dr. Cummings, he took me into his private office and, unlocking a drawer, took out a set of teeth mounted on a rubber plate. This was soon after put on the market and within a year we commenced the use of it, sending our first cases to New York to be vulcanized, and later on procuring a vulcanizer about ten inches in diameter. Two years from the time we began to use it I noticed an unusual change in the alveolar process under these rubber plates, and I have been noticing it ever since. The cause was the non-conductibility of rubber, the retention of undue heat causing undue absorption of the alveolar process. This being the case, I deem it the duty of every dentist to inform his patients of the fact. Rubber is by no means an unmixed evil. It is about as good as anything on the lower jaw, where absorption goes on extensively no matter what is used. The majority of dentists to-day have come into practice since the introduction of vulcanite rubber. If they were instructed at all in the construction of metal plates, the instruction was of such a nature that they did not feel able to make them a success, consequently fell back on the simple process of rubber.

The next thing I want to call your attention to is a matter of much importance, and one which has caused a great deal of discussion, and that is the retention of upper plates. Plates in the earlier days were fitted close to the jaw, palate as well as ridge. No such thing as vacuum cavities were used. After they were introduced, I used them for twenty-five or thirty years. It is all of thirty years since I have used a vacuum cavity in any case whatever, even in heavy continuous gum. I saw in the dental journal recently this statement by a prominent writer with regard to fitting upper dentures: "I know of none who feel that they can rely upon merely fitting a plate over an automatically accurate model." He also says, "Those opposed to suction chambers declare that alleged success with them is due to their acting as relief chambers." Now, every dentist knows that the centre of the palate is hard and unyielding. But how many dentists are there who consider that it is the only portion of the upper jaw which never changes? Your plate is resting and rocking over that hard centre. If an air-chamber is used the anterior and posterior margins rest on the centre and rock. As the alveolar ridge gives way, it is only a question of time when. My practice has been to provide for that change by putting a thin film of wax over the hard centre, extending it back to within one-quarter or perhaps three-sixteenths of an inch of the back of the plate, which I extend farther back than most dentists do. Contrary to what this writer says, "Those opposed to suction chambers declare that alleged success with them is due to their action as relief chambers,"—they do not act as relief chambers. There is another thing in regard to the air-chamber: the patient has to exhaust the air from it, but with the "relief" the plate is simply pressed up. The plate is in close contact with the membrane, and you get all the adhesion that is necessary. In a rubber plate, of course, either scrape it or the impression.

I want to call your attention to another thing, and that is, in flat, ridgeless jaws better suction can be had with metal plates than with rubber. Here is a model of a case the conditions of which were the worst of any case I ever saw, and yet the patient, who had six plates made by different dentists, told me that with this metal plate he often forgot he was wearing artificial teeth.

Here are two models, in general appearance about the same, both of them bad enough, but they are totally unlike in one respect, and are typical cases. One represents ninety-eight per cent. of jaws having the hard centre and requiring the "relief." The other represents two per cent. of jaws having a soft condition of the centre and usually a crevice. These need no relief, but the plate is to be fitted close to the palate.

In the shaping of upper plates, they should be worn as high as possible all around, and always higher over the cuspids than elsewhere, and the gum made fuller. I emphatically object to the use of "canine" as applied to human teeth.

The lower jaw is the troublesome one. I often say to the patient, "Had I no more trouble with lower sets than with uppers, I should be happy." One thing to be guarded against is allowing the lingual margin to be too deep, so that it is lifted by the action of the muscles and loose integuments.

As to the matter of occlusion, there are more failures from faulty occlusions than from any other cause. There may be a perfect-fitting plate, good adhesion, everything right until the jaws close, and then there is trouble. My rule is that none of the six anterior teeth should meet the lower ones; the pressure should be exact on both sides and on the bicuspids and first molar. If there is a second or third molar pitching forward, the upper tooth should not come in contact with it, as the plate would soon be crowded forward.

In regard to continuous gum work, it remains to-day the only perfect denture for full upper sets ever made; the strongest, most durable (when properly made), most natural in appearance, and the only cleanly work used. In 1851 I purchased an office right from John Allen's agent when he came to Boston. A dozen dentists in Boston purchased rights, but had all abandoned it in a year. I have used it continuously ever since. I said if the work was made according to the agent's instructions it would fail. The other dentists followed the instructions and failed, but, seeing where the instructions were faulty, I profited by it and have succeeded. Too much pains cannot be taken in the construction of this work, especially in securing strength, which lies in the metal foundation. The dental laboratories are making the work cheap by using very thin platinum, no reinforcing of the heel, no backing,—an all-important feature.

Patients have little conception of the different conditions of the jaws. While in one case all the conditions are favorable to the wearing of an artificial denture, in another case the conditions are unfavorable, and the patient will never have the satisfaction the other patient has.

In regard to the soft ridge, I always advise its removal, but few patients consent to it.

As to the extraction of certain teeth, my rule is that if it will make the artificial denture more serviceable by all means do it.

DISCUSSION.

*President Faxon.*—I will ask Dr. Guilford to open the discussion.

*Dr. Guilford.*—I hardly like to discuss this paper, because I am afraid I shall spoil the good impression Dr. Haskell has made. He has given some very valuable points based upon his large experience. Dr. Haskell occupies a very unique position in the dental profession, for he is the one man whom I consider to-day the highest authority on this subject we have in America, if not in the world. I pay this tribute to him not in the way of flattery but as a sincere compliment, knowing that he deserves it. When the time comes for me to wear artificial teeth, I hope Dr. Haskell will be alive to make them for me. All the points that have been brought out in the paper are valuable because they have been used and are so intensely practical. If there is one thing harder than another in dentistry, it is becoming skilful in prosthetic work and doing it in such a way as to satisfy yourself and your patient. The country is filled with men who can operate well, but the men who can construct a denture to meet a difficult case are comparatively few. Why is that so? For two reasons, I think. One is that in operative dentistry there have been such wonderful advancements made within the last forty years, advancement at every point, and it has been such attractive work that the majority of men have preferred it and have neglected the other branch. All men want to fill teeth.

They seem to consider it the highest point of attainment in the practice of dentistry. We teachers have to try and impress upon the minds of students that it is a great deal easier to fill teeth than to construct a denture properly. The troubles that come to me in my practice are not those associated with filling, but those related to prosthetic work. There is a great deal of trouble in the peculiar conditions of the mouths. One patient comes with one form of mouth and another with another, and these conditions are constantly varying. If you have sufficient skill and sufficient knowledge, you may make a success of nearly every case, and be considered a very successful prosthetic dentist. Dr. Haskell knows all about it. It is the fifty-seven or fifty-five odd years he has devoted to this work that has made him as eminent as he is to-day in prosthetic work. Nearly all of those before me come from smaller places where more prosthetic work has to be done than in the larger cities. I wish to add two little points to what Dr. Haskell has said. He has told you how to overcome the difficulty of the hard ridge. What are you going to do with the soft ridge? All of you have had cases where an individual has lost four anterior teeth above, and the process has been entirely resorbed. You make a plate from a plaster impression, the patient bites on it and down it comes. I have known cases where four attempts were made to secure a fit under such conditions, and all of them were failures. To overcome the difficulty, take a piece of wax large enough to fill the vacant space between the cuspids and take an impression of that part alone. Chill it, fill the cup with plaster, and reinsert. When the chilled wax comes in contact with the soft ridge it will compress it, while the rest of the roof of the mouth is left perfectly free to be taken with plaster. When the finished plate is inserted and sucked up it will rest as firmly on the compressed tissues in front as upon the hard palate. Sometimes you find mouths in which there is just a single molar remaining, and it is slightly loose with the tissues about it much resorbed. You wish to take a plaster impression. What are you going to do with this tooth? Some will claim that wax is as good as plaster under such circumstances. Others will say, Take your impression in plaster, remove the cup, and cut it out. My plan of meeting the difficulty is to take a piece of French rubber tubing with a hole perhaps the size of a quill, cut off a section long enough to reach from the cervical margin to the widest part of the crown, leaving the occlusal surface entirely free.

Slip it over the tooth and take an impression in plaster. When you take it out the rubber band will disappear. After the impression is poured you will have an exact impression except that the plaster tooth will be larger by the thickness of the rubber band. Take a knife, trim away the excess, and you will have an excellent model. I get a better result in this way than in any other.

*President Faxon.*—I would like to call on Dr. George F. Grant, of Boston.

*Dr. George F. Grant.*—Mr. President, I should like to have time enough to tell you what I think and what I have known of Dr. Haskell's work in prosthetic dentistry. I can simply say, however, that I consider him the greatest exponent of prosthetic dentistry in my time. I have enjoyed hearing him talk. It is a great pleasure, and I have enjoyed it, for I have never had this pleasure before. There are many things that I should like to touch upon. Dr. Haskell commences with the impression, and has gone on to the adjustment of the plate. There are a few things I should like to add on the plate fitting, but time will not permit, so I will come at once to the subject of arranging the teeth in such a manner as to give the best effect as to appearance. I consider an artistic conception of this subject one of the great remedies for what is rapidly becoming a great factor in causing such wide-spread, almost universal, facial disfigurement of the American people. Almost every man who visits the smaller towns, especially of New England, may notice the fearful havoc which the wholesale and inartistic insertion of artificial teeth has wrought in the facial expression.

In our schools it has been my experience that most men will dodge prosthetic dentistry if it is possible for them to do so. This is a very grave mistake. Of course, the operative branches are more attractive to the majority, but I would impress this truth upon your notice. A sound and thorough training in prosthetic work is the best foundation upon which to build a real proficiency in our science.

The schools are somewhat to blame for the neglect of prosthesis. The operative department has a ratio of about twenty teachers to one in the prosthetic department. I believe that there must be a decided reaction in this regard. It is not good practical or business policy to do an ordinary class of work for a low fee. It will really injure everybody and benefit none. The American dentist is likely to lose some of his world-wide prestige unless the standard of prosthetic achievement is elevated to the plane now occupied by the operative field. Prosthetic dentistry is an art which by neglect has fallen almost a quarter-century behind operative dentistry, so that, as a whole, the profession stands as a rather one-sided affair, and the field of prosthetic dentistry presents the wider range of opportunity in the line of advancement. You all know that a piece of poor artificial work is its own condemnation.

As time presses, I will drop only one more hint on the too prevalent mistake in the color of the teeth, especially where a number of teeth are inserted. In an entire denture there should be variety of shading to break the monotony of color. If you have not tried it, the result of a little attempt will be a pleasant surprise.

There are other points to which I hope to be able to call attention, but there is no more time, so that they must be omitted.

*Dr. Isaac J. Weatherbee,* Boston.—I have but a few words to say. That is with reference to the treatment of the soft tissues in the anterior portion of the jaw. Both of the former speakers left it there. Now, this is not proper under any circumstances whatever. The soft tissue should be cut away. A great many cases of that kind come under my notice, and every one has been cut away until I have a firm foundation for my plate to rest on. Then there would not be the least tipping of the plate. To put in a plate over the soft tissue is not proper at all.

*President Faxon.*—Are there any questions any one would like to ask?

*Dr.* ——.—If you had a sound tooth in the jaw, would you leave it there?

*Dr. Weatherbee.*—There is one thing I wish to say and so define my position. There are some dentists who always leave a sound tooth in the upper or lower jaw. I declare most emphatically that the man who will do that thing is a humbug. Why leave a molar in the under jaw? Why do it? It has no business there. It should be removed every time. This idea of leaving one or two teeth is a very absurd idea and should be done away with in the profession.

*Dr.* ——.—If there are two molars, would you leave them?

*Dr. Weatherbee.*—I would extract them at once. Why? Because the denture will settle down and then there would be a bunch on the under jaw. Then, again, these two teeth back of the plate will settle and you will have to make a new plate. Take them out every time. Never be so big a fool as to leave them.

*Dr. George F. Grant.*—I should rather dissent from that, because we have altogether a wrong idea of the permanency of our work in the prosthetic line. Suppose at the end of five or six years the plate should settle, and suppose these teeth were still firm. It is always easier to make another plate, and you have something which you can remove. We are never justified in removing a good, sound tooth from the mouth.

*Dr.* ——.—Suppose there are three molars standing on either side, what would Dr. Weatherbee do?

*Dr. Weatherbee.*—My impression is, if the teeth were absolutely sound and gave assurance that they would never decay, then the lower plate would not drop below. If it were certain they would not decay, I might be tempted to leave them in position, but I have never yet been guilty of the fault of leaving a few teeth, one or two, on either side to hereafter bother the patient. For the sake of the beauty of the lady, and a good conscience on my own part, I think I should remove them.

*President Faxon.*—I have the pleasure of introducing to you, ladies and gentlemen, Dr. Joseph Head, of Philadelphia, whose subject is "Porcelain Inlays."

*Dr. Head,* Philadelphia, Pa.—For the last eight or ten years I have used the high-fusing porcelain that melts at a much higher point than gold, but it must be understood that I do not claim that high-fusing body is better than the low-fusing body, but that it gives better results in my hands. The Jenkins low-fusing body does seem to be permanent and resistant to the fluids of the mouth, but when that porcelain stands the test for five or six years more, we shall be able to speak more advisedly of its merits.

Dr. Jenkins has recently made some comparative tests between his and five other porcelains of the higher-fusing type. These tests tended to prove the superiority of the Jenkins porcelain in density, toughness, and edge strength. Still, as only two experiments with each porcelain were made, the results based on such small data must be considered inconclusive, especially in the light of the hundreds of experiments conducted by Dr. Gilbert, that seem to point to an opposite conclusion.

Nevertheless, in spite of the incongruity of these researches all experimenters in the field of porcelain should receive the thanks of the profession, and it is to be hoped that Dr. Jenkins, Dr. Gilbert, and others will continue their researches and publish them, so that the good points of both high-fusing and low-fusing bodies may be thoroughly revealed.

*President Faxon.*—Many men of many minds means that we all see things from a little different stand-point, hence that argument, agitation, and unrest which is the march of progress. The one whom I shall ask to open the discussion is one who has had practical experience with these bodies in porcelain work, Dr. Charles C. Patten, of Boston.

*Dr. Charles C. Patten.*—I hardly think the little I have to say in regard to my attitude will justify me in appearing before you. The important question in regard to porcelain to-day from my observation is the relative merit of the high- and low-fusing bodies. My experience began with the high-fusing body, and the results that I have obtained were decidedly satisfactory. But I did run up against a few obstacles in the using of the platinum matrix, and was induced to use a gold matrix at times. I have been much pleased with the results obtained with the gold matrix. In regard to the difference in the merit of the high- and low-fusing bodies, I am not fully prepared to discuss that. My experience in low-fusing bodies has been confined to the Jenkins, which are to my mind as nearly perfect as anything which can be secured. The manipulation of it is very simple and easy and the results are good. The fusing of the body in the matrix is simply done and the results are just exactly what you expect. Complaints come from one and another in regard to the lack of stability of the colors, but I think if the work is carefully done and properly shaded when the process of fusing is accomplished, the results will be highly satisfactory and closely approximate the shades that go with the outfit. One little point of difference in regard to the fusing of high and low bodies is the possibility that beginners have difficulty in the porosity of the high-fusing body. That was a matter which bothered me in my early work a good deal, but it is also a matter which is easily overcome by care and experience, and the principal difficulty in that direction is haste. I have never had any of that trouble with the low body. Now, about the only difficulty which I could mention is the tendency for the low-fusing body to assume a globular shape. This seems due to the same reason we find the high-fusing body will become porous,—lack of sufficient care. Do not draw it out before it is fused. Wait until it takes its shape in the matrix, which it will to perfection. The matter of comparison between the high- and low-fusing bodies is of very great interest and importance, and my experience in the high-fusing bodies in contour work of all kinds has been decidedly satisfactory. I fail to see how anybody could ask for anything that would be more so in the application of porcelain in the restoration of corners of the central and lateral incisors and cuspids, or the crowns and proximal surfaces of the bicuspids and molars. The same use can be made of the Jenkins, and permanent results expected if proper care is followed out in the fusing. A great many failures and mistakes are due to a lack of knowledge of the application of the materials at hand. It is absolutely true that all men, and ladies too, as some ladies have taken up this work, with more or less success, cannot become proficient operators in porcelain. Some are more properly fitted and better qualified to pursue this line of work than some others. It is the tendency of our profession to be divided into specialties in the same way the medical profession is becoming divided. We all have a particular taste, and if that taste is allowed to grow and develop unconsciously, we lean in a certain direction by reason of our constitution, and some of those who are pursuing porcelain work will arrive more nearly to perfection than others. Some of us are fitted for crown- and bridge-work, and should make a specialty of that. Ultimately porcelain work will have its true standard, and it has already been accepted as a permanent thing for our profession. I thank you for your kind attention.

*President Faxon.*—I desire to introduce to you Dr. Robert T. Moffatt, of Boston.

*Dr. Robert T. Moffatt.*—The subject of porcelain is a very large one, and I will not take you all through it. I only want to mention one point. That is the difficulty of color. It is a difficulty which probably gives as much trouble as anything in the whole subject. (Illustration.) In making a porcelain filling you will get what will be apparently a perfect match, but after it is set in the cement you will be discouraged to find how much darker it is. It is like buying cloth. We buy some blue serge and try to match that with blue silk and satin ribbon, and they would not look alike, although they were the same shade. There is a different characteristic in each. Now, I am going to take a sample case. (Illustrates.) Take a central incisor with a small labial cavity. I want you to imagine a section cut down through there. On the outer surface of this you have got a translucent layer of enamel. This portion (dentine)

of the tooth is rather opaque. When we make a porcelain filling with the bodies which are now in the market, you get a translucent filling set with an opaque cement, so that the light rays are reflected back to your eye, instead of passing on, as it does through enamel and dentine. Here, when you look at it, it looks dark from the front, but when you move to the side you get a ray of light which strikes the sides of the cavity, which are covered with an opaque layer of cement. It seems to me that the only way to overcome that is to make a body which will have an opaque basis and a translucent enamel. The cement washes out after a short time from the surface of the joint, which seems to improve the match. I can recollect a central incisor which I made in the fall of 1895. I made a corner for it by taking a wax impression of the tooth and making a plaster model, and then building it up with the same material used for porcelain teeth (a true body and enamel, *not glass*). That has been in there for seven years, except for a time when the patient was in the hospital and had it knocked out by a screw gag while undergoing anæsthesia, and in all that time it has been perfect. This joint here, on the labial surface, was practically a perfect fit, as good as could be obtained. The palatal joint was not good, but notwithstanding that, the porcelain corner is satisfactory. I think you will find that in looking at some of these corners they appear all right from one direction, but from another they look entirely different, and I think that the cause is the opacity of the cement used for setting.

### ARTICULATION AND ARTICULATORS.

BY DR. J. ARTHUR ROBINSON, MORRISVILLE, VT.

While bringing to you anything concerning dentistry, I am reminded very forcibly of the old saying about carrying coals to Newcastle. But I am reminded constantly and in every place I may be, whether in Boston, Hartford, where I had the pleasure of attending the Connecticut State Dental meeting, or elsewhere, that there is a lack exhibited by nearly all dentists or makers of artificial plates in the care of articulation. I have given much time and labor to this subject, and, while I do not profess to have reached perfection, I know I am nearer it than some appear to be by the cases to be seen about us every day. I am indebted to the late Dr. Bonwill for a starter in the matter of improvements in articulation. But

Fig. 1.

Fig. 2.

Fig. 3.

I do not learn that he or any one has advocated the idea that the cusps of the posterior teeth are any way but on a level. Though Dr. Bonwill and others have described the line drawn on the cutting edges and the grinding surfaces from front to back as a gradually ascending line to the ramus, still not much has been said of the line drawn in conformity to the grinding surfaces of the posterior teeth, drawing it through from one side to the other. Dr. Bonwill drew this line straight (Fig. 1). Others draw it curved with ends down (Fig. 2). I say it should be a curved line with ends curving up (Fig. 3). I will copy a little from a paper read by myself before the Vermont State Dental Society in 1900, and published in the *Dominion Dental Journal* in June of that year, a goodly part of which was stolen by a Dominion man and read and published as his own the year following.

"I claim the usual relation of the jaws, or rather the two sets of the human teeth, is as a ball and cup. The lower jaw carried either laterally or forward and back will resume its place when carried to its natural position with a motion as though a cup were being placed on the under side of a ball just fitted to it. Take a small straight edge like a pencil, lay it across the lower teeth, and while the buccal cusps touch the pencil the lingual do not. By reversing the pencil to the upper teeth you will find the opposite, showing the idea of the ball and cup, the upper teeth forming the ball and the lower the cup. The line drawn in conformity to the faces or grinding surfaces of the posterior teeth is a curved line, and the arc of a circle varying from a very small one to almost a straight line."

Artificial teeth, if articulated according to this idea, will, when used, come together to a common centre, so to speak, and remain so until again carried to either side as in mastication. If articulated as in Fig. 1, there is nothing to prevent them from sliding from side to side. How about Fig. 2? It is my idea that teeth articulated like that will afford the maker a lot of fun in repairing plates. With teeth set up similar to Fig. 3 there will be a tendency to crowd the upper teeth together, but if like Fig. 2 the plate will invariably crack.

Much might worthily be said on this subject, but owing to lack of time I will not say more.

WALDO E. BOARDMAN, D.M.D.,
*Editor Massachusetts Dental Society.*

## Figures and Tables

**Fig. 1. f1:**
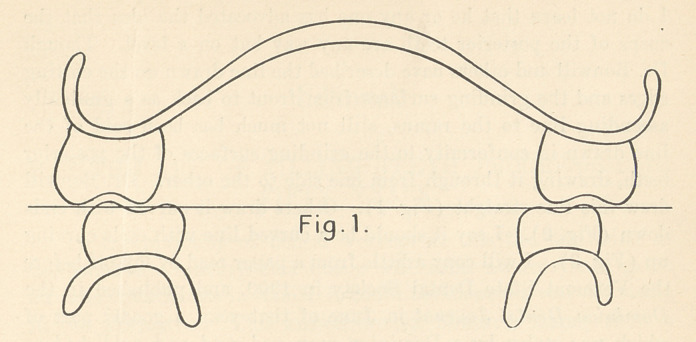


**Fig. 2. f2:**
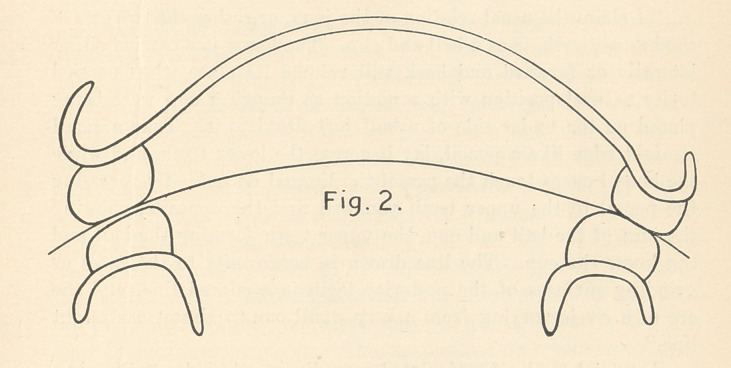


**Fig. 3. f3:**